# ADMET and Solubility Analysis of New 5-Nitroisatine-Based Inhibitors of CDK2 Enzymes

**DOI:** 10.3390/biomedicines11113019

**Published:** 2023-11-10

**Authors:** Przemysław Czeleń, Tomasz Jeliński, Agnieszka Skotnicka, Beata Szefler, Kamil Szupryczyński

**Affiliations:** 1 Department of Physical Chemistry, Faculty of Pharmacy, Collegium Medicum, Nicolaus Copernicus University, Kurpinskiego 5, 85-096 Bydgoszcz, Poland; tomasz.jelinski@cm.umk.pl (T.J.); beatas@cm.umk.pl (B.S.); 2 Faculty of Chemical Technology and Engineering, Bydgoszcz University of Science and Technology, Seminaryjna 3, 85-326 Bydgoszcz, Poland; askot@pbs.edu.pl; 3 Doctoral School of Medical and Health Sciences, Faculty of Pharmacy, Collegium Medicum, Nicolaus Copernicus University, Jagiellońska 13, 85-067 Bydgoszcz, Poland; kamilekszupryk@gmail.com

**Keywords:** isatin, CDK2, competitive inhibition, ADMET, solubility, cosolvation

## Abstract

The development of new substances with the ability to interact with a biological target is only the first stage in the process of the creation of new drugs. The 5-nitroisatin derivatives considered in this study are new inhibitors of cyclin-dependent kinase 2 (CDK2) intended for anticancer therapy. The research, carried out based on the ADMET (absorption, distribution, metabolism, excretion, toxicity) methods, allowed a basic assessment of the physicochemical parameters of the tested drugs to be made. The collected data clearly showed the good oral absorption, membrane permeability, and bioavailability of the tested substances. The analysis of the metabolite activity and toxicity of the tested drugs did not show any critical hazards in terms of the toxicity of the tested substances. The substances’ low solubility in water meant that extended studies tested compounds were required, which helped to select solvents with a high dissolving capacity of the examined substances, such as DMSO or NMP. The use of aqueous binary mixtures based on these two solvents allowed a relatively high solubility with significantly reduced toxicity and environmental index compared to pure solvents to be maintained, which is important in the context of the search for green solvents for pharmaceutical use.

## 1. Introduction

The design of new drugs is not only limited to the search for substances that show pharmacological potential in relation to the chosen biological target, but also requires taking into account other significant properties of potential drugs, such as molecular properties, solubility [1,2,3], absorption [4], metabolism [5], or toxicity [6,7,8], which can significantly affect the practical use of the developed substances. The development of in silico methods allowing these properties to be modelled at a very good level of estimation has contributed to the spread of a significant number of tools that are commonly used in the early phase of drug design, such as ADMETlab2.0 [9], SwissADME [10], pkCSM [11], FAF-Drugs4 [12], and AdmetSAR [13]. Their use allows a significant narrowing of the group of substances considered in in vitro research by excluding chemical compounds with an inappropriate ADMET profile, such as those with properties like the absorption, distribution, metabolism, excretion, and toxicity of considered compounds [4,7,9]. The compounds considered in this work are derivatives of 5-nitroisatin, new inhibitors of cyclin-dependent kinase 2 (CDK2). The overexpression of CDK2, as well as other enzymes belonging to cyclin-dependent kinases (CDKs), is closely associated with the development of cancers. This enzyme plays a crucial role in the regulation of the cell cycle, as well as in the regulation of other enzymes involved in transcription and replication processes [14,15]. Therefore, the development of new inhibitors is crucial in the creation of new effective anti-cancer therapies. The studies carried out so far have allowed a convenient synthesis path to be created, and have shown the significant inhibitory activity of several developed compounds with respect to the active site of the tested enzyme [16]. The further development of the tested compounds as potential drugs requires more knowledge of the physicochemical properties determining their absorption, bioavailability, and toxicity. A significant problem observed in the previous physicochemical analyses is the low solubility of the developed group of inhibitors in water.

Solubility plays a vital role both in the theoretical and practical applications of chemical compounds [1,17]. The capacity to dissolve in a specific solvent has substantial effects on the reactivity, stability, and bioavailability of a compound. In the field of pharmaceuticals, solubility holds significant importance in drug design and formulation [2,18,19,20] and is utilized in many stages of the process of drug manufacturing, ranging from extraction to crystallization. In fact, solvents are so crucial in the pharmaceutical industry that it is even estimated that they make up 80–90% of the overall volume of chemicals employed in drug manufacturing [3]. Insufficient solubility can impose restrictions on a drug’s effectiveness and bioavailability, resulting in escalated expenses and reduced patient adherence. The processes of synthesis, purification, and separation during drug manufacturing can also be hampered by unsatisfactory solubility. Hence, the establishment of efficient and successful techniques for assessing compound solubility holds immense significance [21,22,23,24]. Apart from neat solvents, solvent mixtures, particularly with water, are also widely studied. The so-called cosolvation effect occurs when a specific quantity of a secondary solvent is introduced into the primary solvent, resulting in an augmentation of solubility, which in some cases can be quite spectacular [25,26]. If a specific chemical compound is synthesized for the first time, extensive solubility data are usually not available. Consequently, there is a need to perform initial experiments that would guide future considerations regarding the solubility of an active pharmaceutical ingredient (API). It is also widely accepted that solubility data can be used in theoretical screening procedures which aim to find more efficient solvents by means of different computational methods [27,28].

In this study, several organic solvents were considered for testing the solubility of the studied derivatives. These comprised not only classical organic solvents such as DMSO, DMF, and acetone, but also less popular solvents like 4-formylmorpholine and ethyl lactate. Such a variety of studied solvents is important from the perspective of green chemistry and the search for solvents that are non-toxic, non-flammable, and have a low environmental impact [29,30,31,32]. Among the studied solvents, even the commonly used DMSO can be considered a green solvent [33]. Also, solvents such as 4-formylmorpholine [34], the three studied glymes [35], lactones [36], cyrene [37] and ethyl lactate [38] are commonly considered green. Apart from neat solvents, for the two best-performing systems, binary solvents were also created by mixing these solvents in varying proportions with water. This was conducted in the hope of achieving an even greater solubility of the studied derivatives, as well as a potential reduction in the volume of the used chemicals.

The aim of the presented study was related to obtaining knowledge about the properties of CDK2 inhibitors developed previously [16], which, as it was mentioned earlier, are crucial in creating new forms of drugs. This objective was realized in both a computational manner and by using the ADMET approach, as well as through experimental solubility measurements involving a variety of solvents.

## 2. Materials and Methods

### 2.1. ADMET Analysis

**Figure 1:**
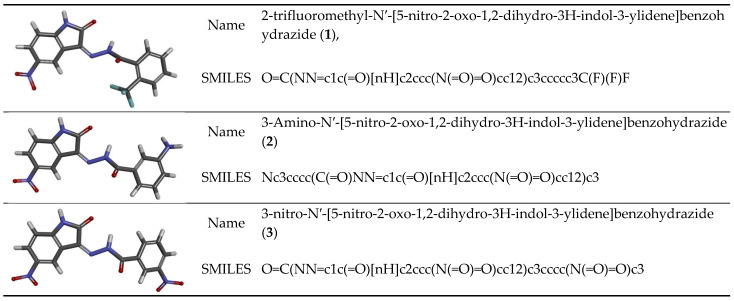
Graphical representations of chemical structures of 5-nitroisatin derivatives together with their SMILES strings.

The subjects of the study of this work are 5-nitroisatin derivatives, which were identified as potential CDK2 inhibitors [39]. The examined chemical compounds are 2-trifluoromethyl-N′-[5-nitro-2-oxo-1,2-dihydro-3H-indol-3-ylidene]benzohydrazide (1), 3-Amino-N′-[5-nitro-2-oxo-1,2-dihydro-3H-indol-3-ylidene]benzohydrazide (2), and 3-nitro-N′-[5-nitro-2-oxo-1,2-dihydro-3H-indol-3-ylidene]benzohydrazide (3) [16]. The evaluation of the descriptors defining molecular properties, absorption [4], metabolism [5,8], and toxicity [5,6] of considered drugs was estimated with the use of ADMETlab 2.0 [9]. The quantitative structure–activity relationship (QSAR) algorithms implemented in used software based on the chemical structures of considered drugs described by SMILES definitions are presented in Figure 1. The method of interpretation and definitions of particular descriptors obtained during the analysis are included in the Results and Discussion sections. The toxicological analysis of solvents was realized with the use of the Solvent Substitution Software Tool, PARIS III [40,41]. The characteristics of the Human Toxicity Potential by Ingestion (*HTPI*) of individual solvents and sets of binary solvents analyzed in this work were obtained based on the values of *LD*_50_ for particular solvents.

(1) $${HTPI}_{i}=\frac{1}{{\left({LD}_{50}\right)}_{i}}$$

Additionally, the values of the Environmental Index (*EI*) were also estimated based on a set of eight environmental impact scores, which included, among others, *HTPI*, terrestrial toxicity potential, and aquatic toxicity potential. In the case of Equation (2), the *σ_j_* is the weight for considered impact *j*, the *φ_i,j_* stands for the value of normalized impact for considered solvent.

(2) $${EI}_{i}=\sum_{j=1}^{8}\sigma_{j}\bar{\varphi_{i,j}}$$

### 2.2. Materials

All reagents and solvents used during synthesis were purchased from Sigma-Aldrich Poland and used without further purification. The highest (≥99%) purity of all used chemicals was required for spectroscopic studies.

The following solvents were used in the solubility experiments: diglyme (CAS: 111-96-6), triglyme (CAS: 112-49-2), tetraglyme (CAS: 143-24-8), 2,4-dimethylphenol (DMP, CAS: 105-67-9), N-methyl-2-pyrrolidone (NMP, CAS: 872-50-4), 4-formylmorpholine (4FM, CAS: 4394-85-8), γ-heptalactone (CAS: 105-21-5), γ-nonanoic lactone (CAS: 104-61-0), ε-caprolactone (CAS: 502-44-3), dimethyl sulfoxide (CAS: 67-68-5), N,N-dimethylformamide (CAS: 68-12-2), dioxane (CAS: 123-91-1), acetone (CAS: 67-64-1), acetonitrile (CAS: 75-05-08), methanol (CAS: 67-56-1), ethanol (CAS: 64-17-5), 1-propanol (CAS: 71-23-8), 1-butanol (CAS: 71-36-3), cyrene (CAS: 53716-82-8), and ethyl(-)-L-lactate (CAS: 687-47-8).

### 2.3. Synthesis

The compounds were prepared by treating 5-nitroisatin with substituted benzoylhydrazine according to the following procedure [16]: equimolar amounts of 5-nitroisatin (0.002 mol) and substituted benzolhydrazine (0.002 mol) were added to 96% ethanol (50 mL) containing 3 drops of glacial acetic acid. The mixture was heated under reflux for 5 h and then cooled to room temperature. The resulting solid was collected via filtration, washed with cold ethanol, and recrystallized from ethanol. All the compounds were analyzed using IR, NMR, and elemental analysis. The *cis*-*trans* isomerism in 5-nitroisatin-based benzoylhydrazines [16] is responsible for the presence of additional signals in NMR spectra.

### 2.4. Characterizations of the New Compounds

2-trifluoromethyl-*Nʹ*-[5-nitro-2-oxo-1,2-dihydro-*3H*-indol-3-ylidene]benzohydrazide (**1**). Yellow solid, yield 87%, m.p. 267.4 °C, d.t. 310.3 °C, IR (ATR), cm^−1^: 3232, 1740, 1690, 1513, 1315. ^1^H NMR (DMSO-d_6_ from TMS) δ (ppm): 13.18 (s, 1H, *cis* form), 12.88 (s, 1H, *trans* isomer), 11.96 (s,1H, *cis* isomer), 11.51 (s, 1H, *trans* isomer), 9.17 (bs, 1H), 8.85 (bs, 1H), 8.32 (m, 2H, *cis* and *trans* isomer), 7,87 (m, 8H, *cis* and *trans* isomer), 7.14 (d, *J* = 8.56 Hz, 1H, *cis* isomer), 7.07 (d, *J* = 8.86 Hz, 1H, *trans* isomer). ^13^C NMR δ (ppm):165.14, 163.46, 150.03, 148.30, 143.31, 142.42, 134.20, 132.92, 131.32, 129.38, 128.26, 126.91, 125.54, 122.82, 122.46, 120.75, 115.38, 112.05, 111.21. C_16_H_9_F_3_N_4_O_4_, Calcd. C, 50.80, H, 2.40, N, 14.81. Found C, 50.74, H, 2.34, N, 14,93.

3-Amino-*Nʹ*-[5-nitro-2-oxo-1,2-dihydro-*3H*-indol-3-ylidene]benzohydrazide (**2**). Yellow solid, yield 92%, d.t. 327.4 °C, IR (ATR), cm^−1^: 3481, 3387, 3204, 1752, 1622, 1514, 1339. *Trans* isomer: ^1^H NMR (DMSO-d_6_ from TMS) δ (ppm): 12.02 (s, 1H), 11.53 (s, 1H), 8.80 (d, *J* = 1.96 Hz, 1H), 8.33 (dd, 1H), 7.24 (m, 1H), 7.18 (s, 1H), 7.09 (m, 2H), 6.85 (m, 1H), 5.48 (bs, 4H). ^13^C NMR δ (ppm): 163.86, 150.04, 149.54, 143.37, 139.06, 134.02, 129.51, 129.06, 122.64, 118.26, 115.89, 114.07, 111.76. *Cis* isomer: ^1^H NMR (DMSO-d_6_ from TMS) δ (ppm): 13.65 (s, 1H), 11.99 (s, 1H), 8.30 (m, 2H), 7.24 (m, 1H), 7.21 (m,3), 6.98 (d, *J* = 8.24 Hz, 1H), 5.48 (bs, 4H). ^13^C NMR δ (ppm): 167.54, 164.07, 149.77, 147.51, 142.27, 136.55, 132.83, 130.12, 127.87, 121.19, 118.78, 116.17, 114.37, 112.99, 111.96. C_15_H_11_N_5_O_4_, Calcd. C, 55.39, H, 3.41, N, 21.53. Found C, 55.43, H, 3.40, N, 21.50.

3-nitro-*Nʹ*-[5-nitro-2-oxo-1,2-dihydro-*3H*-indol-3-ylidene]benzohydrazide (**3**). Yellow solid, yield 92%, m.p. 275.2 °C, d.t. 345.5 °C, IR (ATR), cm^−1^: 3197, 1747, 1684, 1528, 1341. *Trans* isomer: ^1^H NMR (DMSO-d_6_ from TMS) δ (ppm): 12.48 (s, 1H), 11.57 (s, 1H), 8.98 (s, 1H), 8.75 (s, 1H), 8.51 (m, 1H), 8.37 (m, 2H), 7.89 (t, 1H), 7.11 (d, *J* = 8.72 Hz, 1H). ^13^C NMR δ (ppm): 165.24, 150.06, 148.05, 142.37, 135.73, 134.81, 134.02, 130.65, 129.37, 127.17, 122.85, 124.13, 115.61, 111.27. *Cis* isomer: ^1^H NMR (DMSO-d_6_ from TMS) δ (ppm): 13.73 (s, 1H), 12.04 (s, 1H), 8.68 (s, 1H), 8.54 (m, 1H), 8.33 (m, 2H), 8.29 (s, 1H), 7.18 (d, *J* = 8.72 Hz, 1H). ^13^C NMR δ (ppm): 163.65, 148.45, 148.20, 143.41, 134.81, 134.20, 133.63, 131.44, 129.37, 128.30, 123.12, 120.80, 116.42, 112.10. C_15_H_9_N_5_O_6_, Calcd. C, 50.71, H, 2.55, N, 19.71. Found C, 50.86, H, 2.52, N, 19,59.

The ^1^H NMR spectra are presented as Supplementary Materials in Figures S1–S3 for derivatives **1**, **2**, and **3**, respectively.

### 2.5. NMR Measurements

The ^1^H NMR spectra were recorded using an Ascend III spectrometer operating at 400 MHz, from Bruker. Dimethyl sulfoxide was used as a solvent, and tetramethylsilane (TMS) as an internal standard. Chemical shifts (*d*) are reported in ppm relative to TMS and coupling constants (*J*) in Hz.

### 2.6. Elemental Analysis Measurements

The elemental analysis was conducted with a Vario MACRO 11.45–0000, Elemental Analyser System GmbH, operating with the VARIOEL software (version 5.14.4.22).

### 2.7. Calibration Curves

The calibration curves for each derivative were obtained using successful dilutions of initial stock solutions followed by spectrophotometric measurements of the obtained solutions with decreasing concentration. The concentration ranges for the derivatives **1**, **2**, and **3** were 0.0081–0.0405 mg/mL, 0.0077–0.0385 mg/mL, and 0.0068–0.017 mg/mL, respectively. Methanol was used both as a solvent, as well as a reference for spectrophotometric measurements. The analytical wavelengths, corresponding to the highest absorption of the solution, were found to be 315 nm, 321 nm, and 321 nm, respectively, for the three derivatives. The A360 spectrophotometer from AOE Instruments was used for the preparation of the calibration curves. In all three cases, the determination coefficients, R^2^, were found to be 0.999, indicating a satisfactory degree of linearity. The resulting linear equations describing the relationship between the concentration of the solution and the absorbance were found to be A = 55.06 (±0.77)∙C + 0.057 (±0.011), A = 59.02 (±0.65)∙C + 0.041 (±0.001), and A = 84.24 (±0.22)∙C + 0.016 (±0.003) (A—absorbance; C—concentration expressed in mg/mL) for the derivatives 1, 2, and 3, respectively. Three calibration curves were prepared for each derivative and then averaged.

### 2.8. Solubility Determination

A multi-step procedure was employed in order to find the solubility of the studied derivatives in the selected solvents. First of all, the excess amount of the considered compound was added to test tubes containing either neat solvents or their mixtures with water in different proportions, thus forming binary solvents. Saturated solutions were obtained in this way, placed in a Shaker Incubator ES-20/60 from Biosan, and incubated for 24 h at 298.1 K. The temperature adjustment was possible with a 0.1-degree accuracy, and the variance within a 24 h cycle was 0.5 degrees. The samples were simultaneously mixed at 60 rev/min. After incubation, the samples were filtered with the help of plastic syringes equipped with PTFE filters with 0.22 µm pore size. The obtained filtrates were diluted with methanol in another set of test tubes. In order to determine the mole fractions of a solute in the solution, 1 mL of the filtered samples was weighed in 10 mL volumetric flasks using a RADWAG AS 110 R2.PLUS analytical balance. The samples diluted with methanol were measured spectrophotometrically using the same instrument as was used in the case of calibration curves. The spectra were recorded in the wavelength range of 190–500 nm, and the resolution was 1 nm. Methanol was used if further dilution of the sample was required, and it was also used as a reference for calibrating the spectrophotometer. The absorbance values at 315 nm, 321 nm, and 321 nm were taken into account for derivatives **1**, **2**, and **3**, respectively. Based on the calibration curves, the concentration of the solute in the samples was determined together with its mole fraction. Three separate measurements were conducted, and the final values are the results of averaging them.

### 2.9. FTIR Analysis of the Samples

Fourier transform infrared spectroscopy (FTIR) was used to analyze the solid residues remaining after solubility experiments. Before the measurements, the samples were removed from the test tubes and dried on air. A Spectrum Two spectrophotometer from Perkin Elmer with an attenuated total reflection (ATR) device was used in order to obtain the FTIR spectra. Measurements were conducted within the 450–4000 cm^−1^ wavelength range.

## 3. Results and Discussion

### 3.1. ADMET Analysis

**Figure 2:**
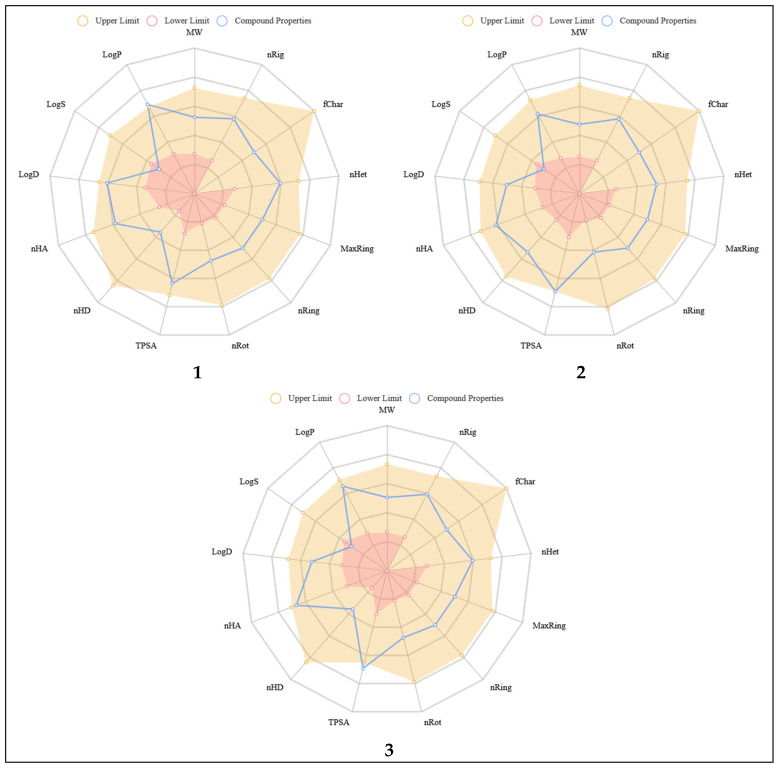
The charts presenting the values of basic descriptors characterizing the molecular properties of considered CDK2 inhibitors (derivatives **1**, **2** and **3**) in relation to the lower and upper limit recommended for substances with pharmacological properties. Molecular weight (MW), number of hydrogen bond acceptors (nHA), number of hydrogen bond donors (nHD), number of rotatable bonds (nRot), number of rings (nRing), number of atoms in the biggest ring (MaxRing), number of heteroatoms (nHet), formal charge (fChar), number of rigid bonds (nRig), Topological Polar Surface Area (TPSA), log of the aqueous solubility (logS), log of the octanol/water partition coefficient (logP), and logP at physiological pH 7.4 (logD) are presented.

The most basic aspects of ADMET analysis, including the values characterizing the physicochemical properties of the considered inhibitors, are presented in Figure 2 and Table 1. The analysis of these outcomes shows that most of the molecular properties of the considered compounds are in the optimal range for pharmaceutical agents. Based on the collected values, it can be unequivocally stated that all analyzed substances meet the basic ADMET rules. In the case of all considered inhibitors, the values of molecular weight, LogP, and quantities of hydrogen bond donors and acceptors meet the requirements of the Lipinski rule [42], which indicates the potentially good absorption and permeability of these compounds. Meeting the requirements of the Pfizer [43] and the GSK [44] rules suggests that the studied substances will have a convenient ADMET profile and relatively low toxicity. In the case of all considered derivatives of 5-nitroisatin, the obtained LogS values confirm the experimental observations indicating the limited solubility of the tested compounds in water. Low water solubility does not disqualify the considered substances as potential drugs; however, it indicates some challenges associated with the development of a form of the drug that enables its absorption and ensures adequate bioavailability. Such a goal can be achieved by using other solvents used in pharmaceutical products. It will also be important to determine the possibility of creating aqueous binary solvents, which are a mixture of water and organic solvents based on the phenomenon of cosolvation. Crossing the body’s barriers and introducing the drug into the bloodstream will enable the further distribution and metabolism of the drug. The presented values of the LogP and LogS coefficients allow the conclusion that the considered compounds exhibit an appropriate balance between lipophilicity and hydrophobicity, ensuring them the appropriate membrane permeability and binding ability to macromolecules such as active sites of biological targets and enzymes involved in cellular transport or drug metabolism. The potential absorption of the considered inhibitors is also described in terms of the values of the human intestinal absorption (HIA), MDCK permeability, Caco-2 Permeability, and Pgp factors. The values presented in Table 1 show that all considered inhibitors should exhibit high HIA. The probability that HIA will be lower than 30% equals nearly zero for all considered inhibitors. The MDCK permeability is the factor based on the Madin−Darby Canine Kidney cells in vitro model created for the screening of membrane permeability and estimation of the blood–brain barrier (BBB). Generally accepted standards for the assessment of these values attribute the high capacity of passive MDCK permeability to compounds described with values greater than 20 × 10^−6^ cm/s. Therefore, all analyzed derivatives can be assigned to this category. Another descriptor of the passive absorption of drugs in the intestine is a model based on human colon adenocarcinoma cell lines (CACO-2) being an adequate equivalent of the intestinal epithelium. This determinant is widely used in the assessment of drug permeability. Compounds characterized by high permeability should have values greater than −5.15 log cm/s. In the case of the tested substances, only inhibitor **1** belongs to this category; the other two derivatives are described by values indicating lower permeability.

A different method of assessing the permeability of compounds is used in the next two descriptors, namely Pgp-inh and Pgp-sub, based on the functionality of P-glycoproteins, which are part of cell membranes and co-create ATP-binding cassette transporters (ABC) [45]. They determine the probability of interaction between the tested drugs and the receptor of the protein in question as an inhibitor or substrate, which may contribute to a significant reduction in the permeability of drugs through membranes. Among the tested 5-nitroisatin derivatives, only in the case of compound **2** was a significant probability of interaction with the above-mentioned receptor as a substrate noted. In the case of drugs that can be administered in an oral form, a significant and frequently used pharmacokinetic parameter is the human oral bioavailability 30% (F (30%)). This value clearly allows the efficiency of introducing the drug into human body fluids to be determined. Values of this descriptor presented in Table 1 describe the probability that oral bioavailability will be smaller than 30%. In the case of all considered inhibitors, the probability of such a situation is equal to zero or nearly zero, which indicates the high oral bioavailability of the considered compounds.

An important factor in the evaluation of new drugs is their metabolism and the potential activity of their metabolites in interaction with the patient’s body. One of the most important roles in the regulation of drug metabolism in the human body is played by cytochrome P450 (CYP) enzymes. The CYP 1–3 enzyme families are involved in at least 80% of drug oxidative processes and 50% of drug elimination from the body, with their most important representatives being Cyp1A2, CYP2C19, CYP2C9, CYP2D6, and CYP3A4 [46,47]. One of the most important factors destabilizing the metabolic processes of drugs regulated by the aforementioned families of enzymes is the phenomenon of inhibition generated by the active forms of drugs and their possible metabolites. An important group of pharmaceuticals with such an ability is agents used in chemotherapy [5,46,47]. This is a frequent source of various types of damage and abnormalities in the functioning of the liver, which is the organ most involved in the discussed drug transformation processes. The carried out ADMET analysis makes it possible to predict the potential properties of the group of inhibitors under consideration. The data in Table 2 illustrate the potential activity of the tested substances as inhibitors and substrates for the most important representatives of cytochrome P450 (CYP) enzymes. When considering derivative **1**, we can see that only in the case of the CYP1A2 enzyme is a significant inhibitory activity of the drug predicted, while in the case of other enzymes, the probability of inhibition is medium or low. The next derivative under consideration, i.e., **2**, shows slightly greater inhibitory activity because its increased level is expected for two enzymes from the considered group, namely CYP1A2 and CYP3A4. derivative 3 is characterized by an increased probability of inhibition of the CYP3A4 enzyme, while in the case of the other considered enzymes, the phenomenon should be negligible. For all considered inhibitors, there is only a medium or very low probability of interaction with the tested group of enzymes as a substrate. Therefore, the tested group of drugs should not significantly interfere with the metabolic processes of other pharmaceuticals metabolized by the analyzed group of enzymes.

An important aspect of the study of chemical substances as potential drugs is the analysis of their toxicity in terms of interaction with the human body. The pharmacological action of a given chemical may be accompanied by a set of undesirable side effects. The studies carried out illustrate a fairly wide spectrum of the potential impact of the tested inhibitors on the human body, and the summary results are presented in Table 3. An important aspect of the potential impact of new drugs is the assessment of their impact on the heart. The determinant of such an impact may be, for example, the ability to block the human ether-a-go-go related gene (hERG) potassium channel. Such drug activity can lead to numerous adverse effects, ranging from cardiac dysfunction to, in extreme cases, a lethal arrhythmia named torsades de pointes (TdP) [48]. The collected data express the probability that the tested substance will exceed the critical value, which is the IC50 for concentrations below 10 µM or the inhibition constant higher than 50% for the limit concentration. In the case of inhibitors **1** and **2**, it can be unequivocally stated that the probability of adverse effects of these substances in this respect is negligible, while in the case of derivative **3**, the probability of blocking activity against ion channels is at a moderate level. Another important aspect of adverse drug activity is their hepatotoxicity (H-HT) and the drug-induced liver injuries associated with it [49,50]. For each of the considered drugs, potential activity was found in the context of interfering with drug metabolism processes carried out by cytochrome P450 (CYP) enzymes in the liver. Such activity may affect the occurrence of undesirable effects, leading to liver damage. The data collected in Table 3 indicate that for each of the considered inhibitors, there is a moderate probability of the occurrence of this type of undesirable effects; the lowest probability was noted for derivative **2**. Another indicator of drug toxicity is the rat oral acute toxicity (ROA) index, referring to the maximum lethal dose in mammals (rats and mice), which is one of the basic indicators of toxicity when evaluating potential drugs. The collected data clearly confirm that for each of the considered derivatives, the probability of exceeding the limit value (ROA > 500mg/kg) is negligible. The considered inhibitors were also analyzed in the context of generating skin sensitization, and the obtained values clearly indicate a low probability of this phenomenon in the case of derivative **1**; a much higher probability of this type of side effect is expected for compounds **2** and **3**. The interaction of the tested drugs with the eyes is determined by the parameters of eye irritation/corrosion (EI/EC). In both cases, the collected data indicate a slight probability of such adverse effects when using the tested derivatives. A common side effect associated with the use of chemotherapeutic drugs is their mutagenic effect; many studies indicate that the implementation of anti-cancer therapies has contributed to secondary cancers [51,52]. Therefore, it is important to determine the risk of using new agents and to be aware of the relevant analyses. The evaluation of the tested compounds indicates that the tested compounds are characterized by a moderate risk of mutagenic activity. Therefore, it would be advisable to carry out research that allows a reliable assessment of this property.

The analysis of the properties of the tested inhibitors indicates a significant deficit related to the low water solubility of the tested compounds. An important step necessary for their potential use as drugs is to determine the most convenient form of their application, which can be a solution based on a solvent suitable for use in pharmaceutical products.

### 3.2. Solubility Determination

When taking into account the solubilities of the three studied derivatives, which are shown in Table 4, a general trend can be easily distinguished. It is derivative **1** that experiences the highest solubility, followed by derivative **2**, with derivative **3** having the lowest solubility. This decreasing trend holds for all studied solvents, with no exceptions. It has to be emphasized that the solubility of derivative **1** is substantially larger than the other two. When comparing the best-performing solvents, this difference is almost 8 times greater and more than 31 times greater than for **2** and **3**, respectively. It is also evident that these two solvents are particularly effective in the dissolution of the studied compounds, namely DMSO and N-methyl-2-pyrrolidone (NMP). In fact, the latter solvent performed best for all three studied derivatives, with x_1_ = 8.06 × 10^−2^, x_2_ = 9.80 × 10^−3^, and x_3_ = 2.44 × 10^−3^. The performance of the second best solvent, namely DMSO, was lower with x_1_ = 7.70 × 10^−2^, x_2_ = 4.63 × 10^−3^, and x_3_ = 6.12 × 10^−4^. Interestingly, the difference between these two solvents was the largest in the case of the derivative characterized by the highest solubility, namely 1. The DMF solvent ranked third in the case of all derivatives (x_1_ = 2.66 × 10^−2^, x_2_ = 3.66 × 10^−3^, x_3_ = 3.69 × 10^−4^). It is also worth mentioning that 4-formylmorpholine (4FM) transpired to be a more effective solvent than acetone, acetonitrile, and dioxane, not to mention the considered alcohols, which were to worst-performing solvents in the studied collection. Also, most classical solvents were outperformed by the three lactones and glymes studied. Even cyrene and ethyl lactate, which were the least effective solubilizers among the studied green solvents, can be regarded as interesting alternatives to many traditional solvents. The three most effective solvents stand out significantly among the tested chemicals, although in the case of derivative **3**, which in general happens to be the least soluble one, these differences are least pronounced. They can be all classified as polar aprotic solvents; however, other solvents of this type, like, e.g., acetone and acetonitrile, are not that effective. A closer inspection reveals that NMP, DMSO, and DMF are characterized by the highest dipole moments among all considered solvents (µ_NMP_ = 4.09 D, µ_DMSO_ = 3.96 D, and µ_DMF_ = 3.86 D), and at least for these three systems, the increase in the dipole moment results in the solubility increase in the studied derivatives.

The two best-performing solvents, namely NMP and DMSO, were also used to create binary solvents with water. This was carried out by mixing the organic solvent and water in different molar proportions with a 0.1-mole step. It can be expected that in such solvent mixtures, a cosolvation effect will occur, which will result in a solubility increase compared to neat solvents. The results of these studies are summarized in Table 5 and Table 6. Two distinct patterns can be observed for DMSO and NMP. In the former case, a cosolvation effect indeed takes place, and the DMSO–water mixture with a composition corresponding to x*_DMSO_ = 0.9 yields a solubility greater than the neat organic solvent. It has to be said, however, that this increase is not very pronounced, and amounts to around 5%. In the case of NMP, however, no cosolvation effect takes place, and the solubility simply increases with the increasing content of the organic solvent. Nonetheless, an important observation is the fact that even small amounts of the organic part in the binary solvent result in quite a substantial solubility of the studied derivatives, and even the x* = 0.1 compositions are responsible for a larger solubility than many neat organic solvents. This is important from an environmental and safety perspective, because it enables the use of small amounts of organic solvents in order to achieve the desired concentration of the active pharmaceutical ingredient. This is particularly important for NMP, as it can hardly be considered a green solvent, although it is still used in the pharmaceutical industry [53,54].

### 3.3. FTIR Analysis of the Samples

**Figure 3:**
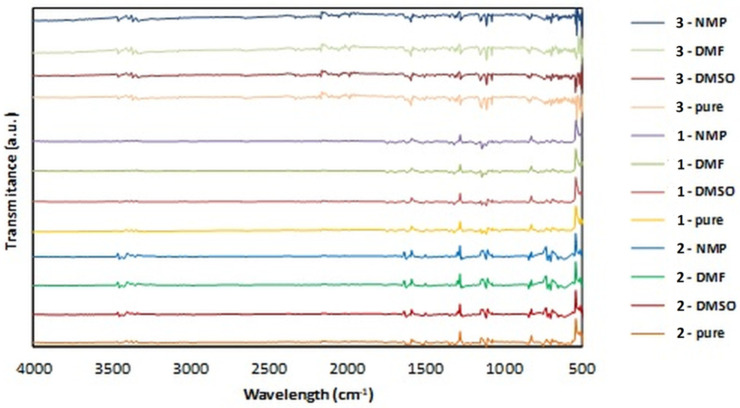
The FTIR spectra of residues of derivatives **1**, **2**, and **3** after solubility measurements in selected solvents. For comparison, the spectra of pure derivatives are provided.

The spectra recorded for solid residues of the studied derivatives obtained after solubility measurements did not reveal any changes compared to the FTIR spectra of pure compounds. There is no shift in the bands, and no additional spectra appear in the samples. This confirms that no changes in the structure of the studied compounds occur in the tested solvents. Only a selected portion of the obtained spectra can be found in Figure 3, in which the FTIR spectra of pure derivatives are shown, together with the spectra of their residues obtained after solubility determination in three solvents, namely DMSO, DMF, and NMP.

The performed solubility analysis of the considered inhibitors has allowed the most efficient solvents to be selected; however, it also seems important to take into account their potential toxicity towards the human body and environmental impact. The PARIS III software was employed for this task. Table 7 includes the values of the Environmental Index (EI) and Human Toxicity Potential by Ingestion (HTPI) index, the values of which were developed on the basis of LD50. The group of solvents with the lowest toxicity includes DMSO, 4FM, ethanol, γ-nonanoic lactone, and triglyme, while the worst characteristics were found in the case of 1-butanol, 1-propanol, acetonitrile, and DMF. The values of HTPI correlate very well with the Environmental Index (EI), characterizing the generalized impact of the considered solvents on the environment. Both indices clearly show that the use of the group of solvents can be highly recommended in pharmacological products. The values are also in good accordance with the literature reporting the use of green solvents.

The conducted research shows that the use of a binary solvent consisting of water and organic solvent enables high solubility with a relatively insignificant content of the organic fraction. The analysis of the HTPI and EI values for the binary systems considered in this work, based on water and DMSO or NMP, is included in Table 8. The accumulated values show that the use of this type of solvent, which still maintains high solubility even for the lowest considered fraction of organic solvent, allows a significant reduction in the toxicity of the studied system by up to 60%, relative to the pure solvent.

## 4. Conclusions

The studies of the basic indicators describing the physicochemical properties of the tested 5-nitroisatin derivatives allow the conclusion that the tested compounds have the correct characteristics in terms of most of the analyzed parameters and meet the requirements of all the basic rules used in the design of drugs. The analysis of the values describing the absorption of the tested drugs, their ability to penetrate cell membranes, and potential bioavailability clearly confirms the good abilities of the tested substances in these respect. The considered inhibitors can probably inhibit only selected enzymes from the cytochrome P450 (CYP) family, such as CYP1A2 and CYP1A4, which may translate into the limited hepatotoxicity expected for the tested group of drugs. The tested inhibitors have good characteristics in terms of rat oral acute toxicity (ROA) index and potential eye irritation or corrosion. Many more warning signals were observed in the case of the potential carcinogenicity of the tested drugs, which, unfortunately, is quite a common side effect among chemotherapeutic drugs. We must be aware that the presented data and the conclusions drawn from them are based on the results of modeling, which is able to largely predict the discussed properties; however, further consideration of the tested compounds as potential drugs requires the experimental confirmation of these properties. The requirement of drugs to have a very low aqueous solubility necessitated the search for new pharmaceutical solvents, which could be used at various stages of drug manufacturing. The research conducted allowed an extensive set of data to be collected, clearly indicating a wide spectrum of solvents resulting in satisfactory solubility. Among the studied compounds, derivative **1** was found to have the best solubility in all studied systems. When taking into account the tested solvents, the best effects were noted in the case of DMSO, NMP, and DMF. An important aspect of the conducted research is the observation that the use of binary solvents based on water and DMSO or NMP, even with a relatively low share of the organic fraction, ensures very good solubility, several times higher than that obtained with other tested solvents. The use of such systems significantly reduces the toxicity and environmental effects of the tested mixtures. The obtained collection of solubility data can guide other authors in their research and lay the foundation for future solubility modeling and screening regarding the studied compounds.

## Figures and Tables

**Table 1:** The values of descriptors characterizing molecular and absorption properties of considered drugs. For Pgp-inh/sub, HIA and F (30%) values describe the probability of meeting the assumed boundary conditions for selected parameters, within the range of 0 to 1. Classification of values is as follows, 0–0.3: excellent; 0.3–0.7: medium; 0.7–1.0: poor.

Property	UNIT	1	2	3
LogS	log mol/dm^3^	−4.831	−4.83	−4.806
LogD	log mol/dm^3^	2.661	2.021	2.122
LogP	log mol/dm^3^	3.15	2.29	2.635
TPSA	Å^2^	113.7	139.72	156.84
MW	g/mol	378.06	325.08	355.06
Pgp-inh	-	0.007	0.002	0.007
Pgp-sub	-	0.016	0.635	0.01
HIA	-	0.012	0.027	0.043
F(30%)	-	0.001	0.002	0
Caco-2	log cm/s	−5.011	−5.585	−5.349
MDCK	cm/s	4.80 × 10^−5^	2.27 × 10^−5^	0.000279072

**Table 2:** The metabolism of considered drugs by enzymes from the human cytochrome P450 group. Presented values describe the probability of being a substrate or inhibitor of considered enzymes in the range of 0 to 1. Category 0: non-substrate/non-inhibitor; Category 1: substrate/inhibitor.

Enzyme	Probability		
	1	2	3
CYP1A2-inh	0.831	0.739	0.555
CYP1A2-sub	0.684	0.1	0.104
CYP2C19-inh	0.489	0.211	0.265
CYP2C19-sub	0.096	0.062	0.062
CYP2C9-inh	0.566	0.403	0.338
CYP2C9-sub	0.691	0.278	0.501
CYP2D6-inh	0.02	0.002	0.011
CYP2D6-sub	0.136	0.15	0.133
CYP3A4-inh	0.392	0.708	0.74
CYP3A4-sub	0.154	0.096	0.069

**Table 3:** Toxicity characteristics of the considered 5-nitroisatin derivatives. Presented values describe the probability of being toxic within the range of 0 to 1. Classification of values is as follows. 0–0.3: excellent; 0.3–0.7: medium; 0.7–1.0: poor.

Property	Probability		
	1	2	3
hERG	0.109	0.205	0.514
H-HT	0.611	0.328	0.561
ROA	0.28	0.117	0.105
SkinSen	0.23	0.508	0.544
Carcinogenicity	0.584	0.767	0.793
EC	0.003	0.003	0.003
EI	0.033	0.217	0.183

**Table 4:** The solubility of derivatives **1**, **2**, and **3** in neat solvents at 298.1 K. Values are expressed as mole fractions (x) and concentrations (c, mg/mL). Standard deviation values are given in parentheses.

Solvent	1		2		3	
	x	c (mg/mL)	x	c (mg/mL)	x	c (mg/mL)
**diglyme**	9.43 (0.90) × 10^−4^	2.52 (0.24)	5.22 (0.10) × 10^−4^	1.19 (0.02)	2.08 (0.07) × 10^−4^	0.52 (0.02)
**triglyme**	4.48 (0.06) × 10^−4^	0.95 (0.01)	2.34 (0.06) × 10^−4^	0.42 (0.01)	1.17 (0.05) × 10^−4^	0.23 (0.01)
**tetraglyme**	2.60 (0.17) × 10^−4^	4.62 (0.30)	1.20 (0.02) × 10^−3^	1.79 (0.03)	2.67 (0.09) × 10^−4^	0.43 (0.01)
**DMP**	2.96 (0.11) × 10^−4^	0.94 (0.03)	1.05 (0.03) × 10^−4^	0.29 (0.01)	0.27 (0.01) × 10^−4^	0.08 (<0.01)
**NMP**	8.06 (0.25) × 10^−2^	2.65 (0.07) × 10^2^	9.80 (0.13) × 10^−3^	3.27 (0.05) × 10^1^	2.44 (0.07) × 10^−3^	8.99 (0.25)
**4FM**	1.88 (0.04) × 10^−3^	7.22 (0.16)	1.06 (0.04) × 10^−3^	3.47 (0.12)	2.65 (0.11) × 10^−4^	0.94 (0.04)
**γ-heptalactone**	5.92 (0.08) × 10^−4^	1.78 (0.02)	4.25 (0.06) × 10^−4^	1.09 (0.02)	1.59 (0.04) × 10^−4^	0.44 (0.01)
**γ-nonanoic lactone**	4.81 (0.06) × 10^−4^	1.16 (0.01)	3.46 (0.07) × 10^−4^	0.71 (0.01)	1.26 (0.05) × 10^−4^	0.28 (0.01)
**ε-caprolactone**	4.42 (0.09) × 10^−4^	1.60 (0.03)	3.25 (0.09) × 10^−4^	1.01 (0.03)	1.02 (0.02) × 10^−4^	0.34 (0.01)
**cyrene**	4.37 (0.09) × 10^−4^	1.62 (0.03)	2.36 (0.04) × 10^−4^	0.75 (0.01)	0.83 (0.01) × 10^−4^	0.29 (<0.01)
**ethyl lactate**	3.75 (0.10) × 10^−4^	1.25 (0.03)	1.95 (0.04) × 10^−4^	0.56 (0.01)	0.57 (0.01) × 10^−4^	0.18 (<0.01)
**DMSO**	7.70 (0.11) × 10^−2^	3.27 (0.04) × 10^2^	4.63 (0.14) × 10^−3^	2.14 (0.07) × 10^1^	6.12 (0.10) × 10^−4^	3.09 (0.05)
**DMF**	2.66 (0.55) × 10^−2^	1.19 (0.02) × 10^2^	3.66 (0.06) × 10^−3^	1.53 (0.02) × 10^1^	3.69 (0.05) × 10^−4^	1.69 (0.02)
**dioxane**	9.21 (0.21) × 10^−4^	4.17 (0.09)	2.49 (0.05) × 10^−4^	0.96 (0.02)	0.74 (0.01) × 10^−4^	0.31 (0.01)
**acetone**	8.38 (0.16) × 10^−4^	4.39 (0.08)	2.00 (0.09 × 10^−4^	0.90 (0.04)	0.43 (0.01) × 10^−4^	0.21 (0.01)
**acetonitrile**	2.76 (0.12) × 10^−4^	2.02 (0.09)	0.96 (0.03) × 10^−4^	0.61 (0.02)	0.25 (<0.01) × 10^−4^	0.17 (<0.01)
**methanol**	1.60 (0.05) × 10^−4^	1.51 (0.05)	0.39 (0.01) × 10^−4^	0.31 (0.01)	0.07 (<0.01) × 10^−4^	0.06 (<0.01)
**ethanol**	1.26 (0.04) × 10^−4^	0.82 (0.03)	0.26 (0.01) × 10^−4^	0.15 (0.01)	0.05 (<0.01) × 10^−4^	0.03 (<0.01)
**1-propanol**	1.19 (0.05) × 10^−4^	0.61 (0.03)	0.23 (0.01) × 10^−4^	0.10 (<0.01)	0.04 (<0.01) × 10^−4^	0.02 (<0.01)
**1-butanol**	1.11 (0.04) × 10^−4^	0.47 (0.02)	0.21 (0.01) × 10^−4^	0.07 (<0.01)	0.04 (<0.01) × 10^−4^	0.02 (<0.01)

**Table 5:** The solubility of derivatives **1**, **2**, and **3** in mixtures of DMSO and water at 298.1 K. Values are expressed as mole fractions (x) and concentrations (c, mg/mL). Standard deviation values are given in parentheses. In the first column, x^*^_DMSO_ denotes the mole fractions of DMSO in solute-free mixtures with water.

x*_DMSO_	1		2		3	
	x	c (mg/mL)	x	c (mg/mL)	x	c (mg/mL)
**0.1**	5.27 (0.10) × 10^−3^	8.80 (0.16) × 10^1^	2.11 (0.02) × 10^−4^	3.22 (0.02)	0.52 (<0.01) × 10^−4^	0.86 (0.01)
**0.2**	1.47 (0.02) × 10^−2^	1.79 (0.02) × 10^2^	5.76 (0.06) × 10^−4^	6.95 (0.07)	1.21 (0.01) × 10^−4^	1.58 (0.01)
**0.3**	2.25 (0.03) × 10^−2^	2.19 (0.02) × 10^2^	1.08 (0.01) × 10^−3^	1.08 (0.01) × 10^1^	2.29 (0.01) × 10^−4^	2.48 (0.01)
**0.4**	3.25 (0.05) × 10^−2^	2.61 (0.03) × 10^2^	1.69 (0.02) × 10^−3^	1.43 (0.01) × 10^1^	3.57 (0.03) × 10^−4^	3.30 (0.03)
**0.5**	4.46 (0.08) × 10^−2^	3.05 (0.04) × 10^2^	2.46 (0.02) × 10^−3^	1.84 (0.01) × 10^1^	4.59 (0.04) × 10^−4^	3.75 (0.03)
**0.6**	5.69 (0.15) × 10^−2^	3.36 (0.06) × 10^2^	3.18 (0.03) × 10^−3^	2.10 (0.02) × 10^1^	5.45 (0.03) × 10^−4^	3.95 (0.02)
**0.7**	6.72 (0.14) × 10^−2^	3.56 (0.06) × 10^2^	4.01 (0.05) × 10^−3^	2.41 (0.03) × 10^1^	6.09 (0.03) × 10^−4^	4.01 (0.02)
**0.8**	7.60 (0.10) × 10^−2^	3.63 (0.03) × 10^2^	4.68 (0.03) × 10^−3^	2.54 (0.02) × 10^1^	6.47 (0.04) × 10^−4^	3.85 (0.02)
**0.9**	8.00 (0.14) × 10^−2^	3.55 (0.05) × 10^2^	4.86 (0.08) × 10^−3^	2.41 (0.04) × 10^1^	6.51 (0.04) × 10^−4^	3.54 (0.02)
**1.0**	7.70 (0.11) × 10^−2^	3.27 (0.04) × 10^2^	4.63 (0.14) × 10^−3^	2.14 (0.07) × 10^1^	6.12 (0.07) × 10^−4^	3.09 (0.03)

**Table 6:** The solubility of derivatives **1**, **2**, and **3** in mixtures of NMP and water at 298.1 K. Values are expressed as mole fractions (x) and concentrations (c, mg/mL). Standard deviation values are given in parentheses. In the first column, x^*^_NMP_ denotes the mole fractions of NMP in solute-free mixtures with water.

x*_NMP_	1		2		3	
	x	c (mg/mL)	x	c (mg/mL)	x	c (mg/mL)
**0.1**	3.86 (0.05) × 10^−3^	5.66 (0.07) × 10^1^	4.43 (0.05) × 10^−4^	5.75 (0.06)	1.12 (0.01) × 10^−4^	1.58 (0.02)
**0.2**	1.05 (0.01) × 10^−2^	1.11 (0.01) × 10^2^	1.23 (0.01) × 10^−3^	1.21 (0.01) × 10^1^	3.05 (0.03) × 10^−4^	3.29 (0.03)
**0.3**	1.98 (0.04) × 10^−2^	1.61 (0.03) × 10^2^	2.32 (0.03) × 10^−3^	1.82 (0.02) × 10^1^	5.74 (0.10) × 10^−4^	4.94 (0.08)
**0.4**	3.09 (0.04) × 10^−2^	2.05 (0.02) × 10^2^	3.53 (0.04) × 10^−3^	2.33 (0.02) × 10^1^	8.92 (0.10) × 10^−4^	6.49 (0.07)
**0.5**	5.43 (0.07) × 10^−2^	2.48 (3.03) × 10^2^	5.24 (0.06) × 10^−3^	2.95 (0.03) × 10^1^	1.24 (0.01) × 10^−3^	7.72 (0.07)
**0.6**	5.77 (0.07) × 10^−2^	2.72 (0.03) × 10^2^	6.71 (0.08) × 10^−3^	3.30 (0.04) × 10^1^	1.60 (0.02) × 10^−3^	8.71 (0.11)
**0.7**	6.87 (0.11) × 10^−2^	2.85 (0.03) × 10^2^	7.95 (0.08) × 10^−3^	3.45 (0.04) × 10^1^	1.99 (0.02) × 10^−3^	9.56 (0.10)
**0.8**	7.76 (0.08) × 10^−2^	2.94 (0.03) × 10^2^	8.96 (0.09) × 10^−3^	3.59 (0.04) × 10^1^	2.29 (0.02) × 10^−3^	1.02 (0.01) × 10^1^
**0.9**	7.96 (0.10) × 10^−2^	2.82 (0.03) × 10^2^	9.53 (0.11) × 10^−3^	3.49 (0.04) × 10^1^	2.40 (0.03) × 10^−3^	9.71 (0.12)
**1.0**	8.06 (0.25) × 10^−2^	2.65 (0.07) × 10^2^	9.80 (0.13) × 10^−3^	3.27 (0.04) × 10^1^	2.44 (0.06) × 10^−3^	8.99 (0.25)

**Table 7:** The values of the Human Toxicity Potential by Ingestion (HTPI) index and the Environmental Index (EI) of solvents used in the solvation of the studied drugs.

Solvent	HTPI	EI
DMSO	0.1300	0.2600
4FM	0.2535	0.5080
ethanol	0.2680	0.5540
γ-nonanoic lactone	0.2860	0.6440
triglyme	0.2910	0.5830
acetone	0.3260	0.6620
diglyme	0.3495	0.6990
methanol	0.3675	0.7420
tetraglyme	0.3355	0.7640
ε-caprolactone	0.4405	0.8830
NMP	0.4830	0.9730
dioxane	0.4500	0.9840
γ-heptalactone	0.5500	1.1100
24DMP	0.5900	1.3100
acetonitryl	0.7650	1.9000
1-propanol	1.0100	2.0900
DMF	0.6750	2.1600
1-butanol	2.3900	4.8600

**Table 8:** The values of the Human Toxicity Potential by Ingestion (HTPI) index and the Environmental Index (EI) of binary solvents created by DMSO and NMP with water, x*_DMSO/NMP_ denotes the mole fractions of DMSO ore NMP in solute-free mixtures with water, % HTPI x_1.0 DMSO/NMP_ denotes percentage HTPI of binary solvent relative to the pure organic one.

x*_DMSO_	HTPI	% HTPI x_1.0 DMSO_	EI	x*_NMP_	HTPI	% HTPI x_1.0 NMP_	EI
**0.1**	0.0490	37.7	0.0981	0.1	0.1890	39.1	0.3820
**0.2**	0.0725	55.8	0.1450	0.2	0.2840	58.8	0.5720
**0.3**	0.0881	67.8	0.1760	0.3	0.3420	70.8	0.6890
**0.4**	0.0993	76.4	0.1990	0.4	0.3820	79.1	0.7690
**0.5**	0.1080	83.1	0.2150	0.5	0.4100	84.9	0.8260
**0.6**	0.1140	87.7	0.2280	0.6	0.4320	89.4	0.8700
**0.7**	0.1190	91.5	0.2390	0.7	0.4490	93.0	0.9040
**0.8**	0.1240	95.4	0.2470	0.8	0.4630	95.9	0.9310
**0.9**	0.1270	97.7	0.2540	0.9	0.4740	98.1	0.9540
**1.0**	0.1300	100.0	0.2600	1.0	0.4830	100.0	0.9730

## Data Availability

All data collected for this work are included in the article and supplementary materials.

## Supplementary Materials

The following supporting information can be downloaded at: https://www.mdpi.com/article/10.3390/biomedicines11113019/s1.
