# Supplementary material for: ADMET and Solubility Analysis of New 5-Nitroisatine-Based Inhibitors of CDK2 Enzymes
Source: Biomedicines. 2023 Nov 10;11(11):3019. doi: 10.3390/biomedicines11113019 (PMC10669656; doi:10.3390/biomedicines11113019)
Supplement: Supplementary file 1 [file biomedicines-11-03019-s001.zip › biomedicines-2705459-supplementary.pdf]

ADMET and Solubility Analysis of New 5-Nitroisatine-Based Inhibitors of CDK2 Enzymes

Przemysław Czeleń [1,*], Tomasz Jeliński [1], Agnieszka Skotnicka [2], Beata Szefler [1] and Kamil Szupryczyński [3]

1 Department of Physical Chemistry, Faculty of Pharmacy, Collegium Medicum, Nicolaus Copernicus
University, Kurpinskiego 5, 85-096 Bydgoszcz, Poland; tomasz.jelinski@cm.umk.pl (T.J.); beatas@cm.umk.pl (B.S.)
2 Faculty of Chemical Technology and Engineering, Bydgoszcz University of Science and Technology,
Seminaryjna 3, 85-326 Bydgoszcz, Poland; askot@pbs.edu.pl
3 Doctoral School of Medical and Health Sciences, Faculty of Pharmacy, Collegium Medicum, Nicolaus Copernicus University, Kurpinskiego 5, 85-096 Bydgoszcz, Poland; kamilekszupryk@gmail.com
* Correspondence: przemekcz@cm.umk.pl

Supplementary materials

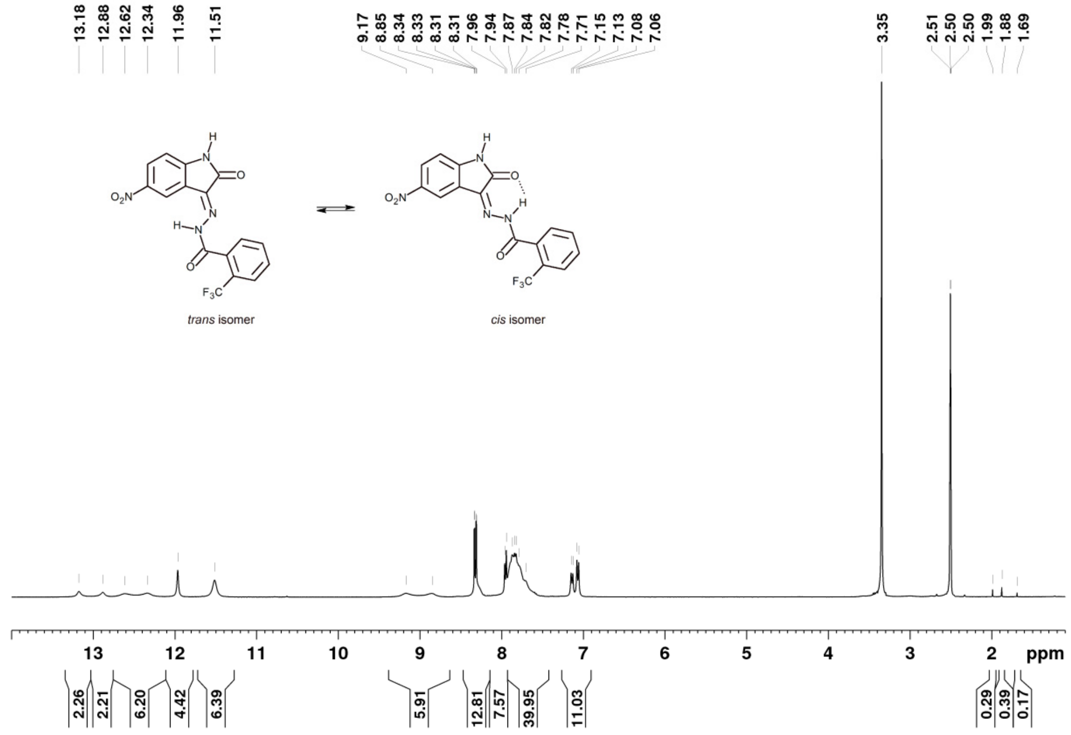

Figure s1. The ^{1}H NMR spectrum (400 MHz) of 2-trifluoromethyl-*N'*-[5-nitro-2-oxo-1,2-dihydro-*3H*-indol-3-ylidene]benzohydrazide (1) in DMSO-d_6.

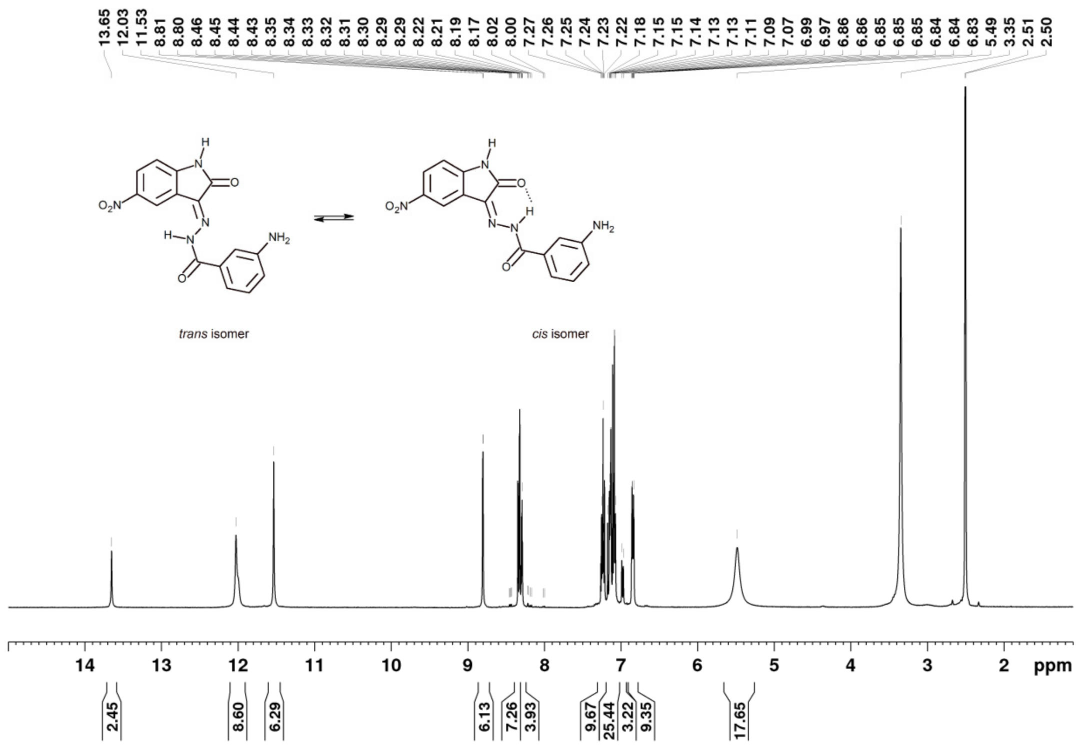

Figure s2. The ^{1}H NMR spectrum (400 MHz) of 3-Amino-*N′*-[5-nitro-2-oxo-1,2-dihydro-*3H*-indol-3-ylidene]benzohydrazide (2) in DMSO-d_6.

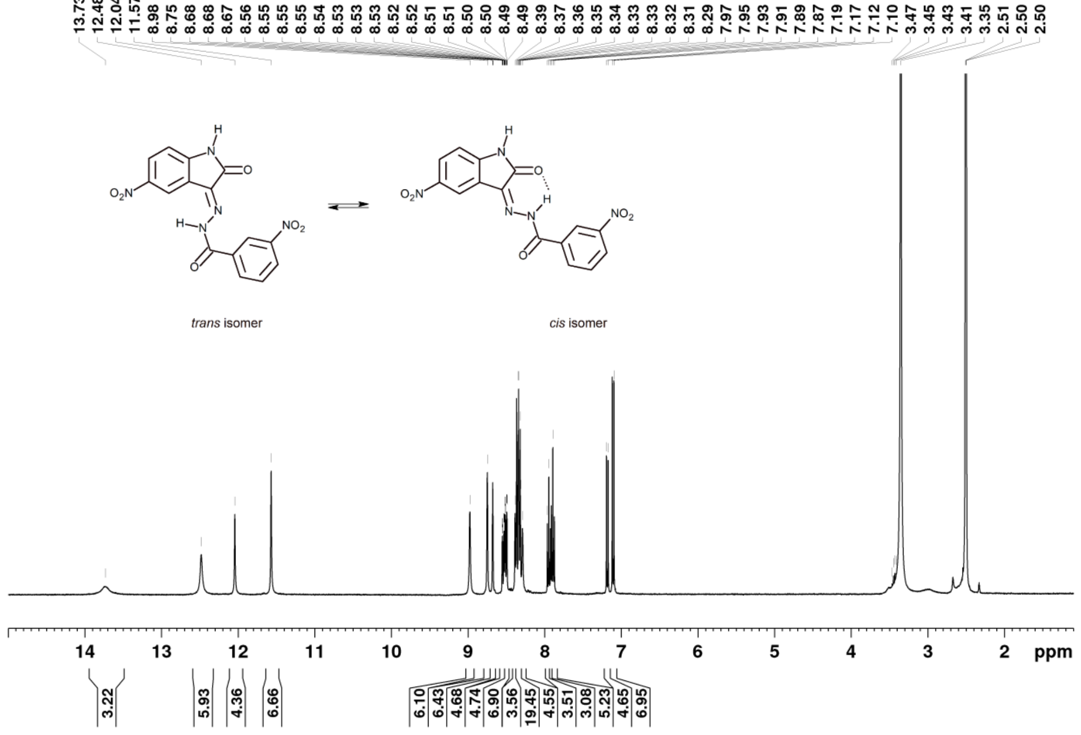

Figure s3. The 1H NMR spectrum (400 MHz) of 3-nitro-*N′*-[5-nitro-2-oxo-1,2-dihydro-*3H*-indol-3-ylidene]benzohydrazide (3) in DMSO-d_6.
